# A Longitudinal Study on Dental Caries Focusing on Long-Term Breastfed Children in Japan

**DOI:** 10.3390/nu17243846

**Published:** 2025-12-09

**Authors:** Masatoshi Otsugu, Yusuke Mikasa, Maika Kadono, Katsura Matsunami, Motomi Nakamura, Yuko Ohno, Takafumi Kato, Kazuhiko Nakano

**Affiliations:** 1 Department of Pediatric Dentistry, Osaka University Graduate School of Dentistry, Osaka 565-0871, Japan; mikasa.yusuke.dent@osaka-u.ac.jp (Y.M.); u214134e@alumni.osaka-u.ac.jp (M.K.); nakano.kazuhiko.dent@osaka-u.ac.jp (K.N.); 2 Toyonaka City Public Health Center, Osaka 561-0881, Japan; katsura.matsunami@city.toyonaka.osaka.jp (K.M.); boshihoken@city.toyonaka.osaka.jp (M.N.); 3 Department of Health Sciences, Osaka University Graduate School of Medicine, Osaka 565-0871, Japan; ohno@sahs.med.osaka-u.ac.jp; 4 Department of Oral Physiology, Osaka University Graduate School of Dentistry, Osaka 565-0871, Japan; kato.takafumi.dent@osaka-u.ac.jp

**Keywords:** breastfeeding, dental caries, postnatal class, mental health, longitudinal analysis

## Abstract

**Background/Objectives:** Although the relationship between prolonged breastfeeding and early childhood caries remains unclear, dentists must manage children’s oral health while respecting mothers’ chosen feeding practices and providing information on maintaining oral health as long as possible. This longitudinal study was performed to investigate the occurrence of dental caries and identify associated factors, with particular attention to maternal condition and oral-health-related support in long-term breastfed children. **Methods:** Of 6746 children aged 42 months in Toyonaka City, Japan, 1210 who had been breastfed for at least the first 18 months were enrolled. Participants underwent oral examinations and anthropometric measurements at 18 and 42 months of age. In addition, a self-administered questionnaire was distributed to families when the child was 18 months old. Logistic regression analysis was used to evaluate risk factors for dental caries, with caries occurrence at 42 months as the dependent variable. **Results:** Overall, 24.3% of the children had experienced dental caries at 42 months. Logistic regression analysis identified several significant factors associated with caries occurrence: birth order (*p* < 0.001), snacking frequency (*p* = 0.038), Cariostat^®^ caries-risk test results (*p* < 0.001), and wake-up time (*p* = 0.015) among child factors, and parental exhaustion (*p* = 0.041) and participation in postnatal oral health classes (*p* = 0.005) among maternal factors. **Conclusions:** Waking habits in early childhood, maternal psychological condition, and participation in postnatal oral health instruction were significantly associated with dental caries occurrence over time among long-term breastfed children.

## 1. Introduction

Breastfeeding is one of the most effective ways to ensure a child’s health and survival [1] and may have lasting effects throughout an individual’s life course [2]. Breast milk is the ideal food for infants and provides benefits for both the child and the mother [1]. Breastfed children tend to perform better on intelligence tests, and they are less likely to become overweight or obese or to develop inflammatory bowel disease, allergic rhinitis, asthma, or diabetes later in life [1,3,4]. Breastfeeding women also have a reduced risk of breast and ovarian cancers as well as metabolic and cardiovascular diseases [1,5,6]. Therefore, the World Health Organization (WHO) recommends breastfeeding up to 2 years of age or beyond [7].

Conversely, prolonged breastfeeding can be a risk factor for early childhood caries (ECC), although its definition remains inconsistent across studies [8,9,10]. Some recent systematic reviews have shown that breastfeeding beyond 12 months of age is associated with severe ECC [8,9], and another concluded that breastfeeding beyond 24 months could increase the risk of ECC [10]. The International Association of Paediatric Dentistry has recommended against breastfeeding and bottle use beyond 12 months to prevent ECC [11]. Conversely, a recent cohort study found that exclusive breastfeeding for 6–17 months provides a protective effect against ECC [12]. Thus, the relationship between prolonged breastfeeding and ECC remains “inconclusive and equivocal” [12].

Nevertheless, dentists must respect mothers’ chosen feeding practices while managing children’s oral health and providing guidance on how to maintain oral health, allowing children to enjoy the benefits of breast milk for as long as possible. Therefore, further research focusing on the characteristics of long-term breastfed children is warranted, particularly from a maternal perspective. Oral-health-related support for caregivers has been reported to be an effective dental approach during the breastfeeding period and can contribute to infant oral health [13,14]. Such support may help prevent ECC in many children, regardless of individual lifestyle or family background. At the same time, emerging evidence suggests that maternal psychological well-being may influence the development of ECC in children [15]. However, the relationship between caregiver-oriented oral health support and ECC among long-term breastfed children has not yet been clarified.

In this longitudinal study, we investigated the occurrence of dental caries in children who were breastfed for at least 18 months, identified associated factors (particularly those related to maternal condition and oral-health-related support), and aimed to deepen our understanding of oral health in long-term breastfed children.

## 2. Materials and Methods

### 2.1. Study Participants

**Figure 1:**
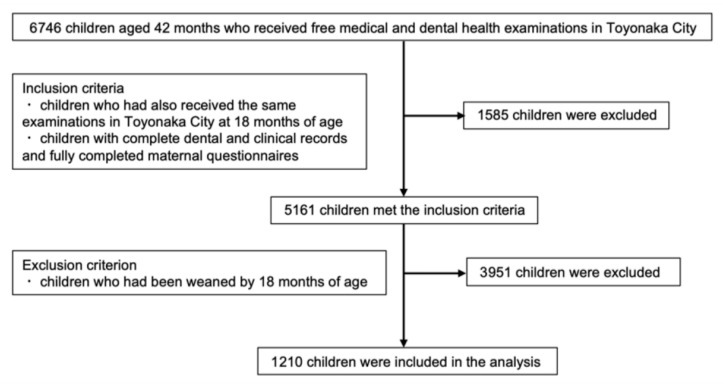
Flow diagram of participant selection in the present study.

Participants were selected from 6746 children who received free medical and dental health examinations at 42 months of age between April 2018 and March 2020 in Toyonaka City, an urban municipality in Osaka Prefecture, Japan. The city provides comprehensive maternal and child health programs, including postnatal oral health education. Of these children, 5161 met the inclusion criteria, which required that they had also undergone the same examinations at 18 months of age, with complete dental and clinical records and fully completed maternal questionnaires. Subsequently, 3951 children were excluded because they had been weaned by 18 months of age. Ultimately, 1210 children aged 42 months who had continued breastfeeding for at least the first 18 months were included in the analysis (Figure 1). Informed consent was obtained from all participants’ guardians through an online opt-out procedure. Information about the study was posted on the official website of Toyonaka City. Guardians were informed that they could withdraw their child’s data at any time by contacting the research office by telephone. No guardians opted out; therefore, no participants were excluded based on the consent process.

### 2.2. Clinical Examination

The physical examinations included anthropometric measurements such as height and weight at 18 months of age. Oral examinations assessed the number of erupted teeth (at 18 months), the number of decayed teeth (at 18 months), and the number of decayed, missing, and filled primary teeth (dmft) per child (at 18 and 42 months). Examinations were conducted by visual inspection with a dental mirror under proper lighting, following the criteria established by the WHO [16]. The number of erupted teeth at 18 months was categorized into three groups based on the typical eruption order of primary teeth among Japanese children: ≤12 teeth, 13–16 teeth, and ≥17 teeth [17]. All oral examinations were performed by dentists from the Toyonaka Dental Association.

### 2.3. Bacteriological Assessment

The bacteriological status was assessed at 18 months of age using Cariostat^®^ (Dentsply Sirona, Tokyo, Japan) to evaluate caries risk [18]. Dental plaque was collected from the buccal surfaces of the maxillary teeth with a cotton swab. The swab was then immersed in the test medium and incubated at 37 °C for 48 h. After incubation, the color of the medium changed from blue to green, yellow-green, or yellow, depending on the acidity of the sampled plaque. According to the color chart, the results were classified into four categories: (−: blue), (+: green), (++: yellow-green), and (+++: yellow), indicating increasing levels of acid production by cariogenic bacteria [18].

### 2.4. Questionnaire

A self-administered questionnaire was distributed to mothers and collected during the 18-month examinations. This paper-based questionnaire included 10 items regarding the children: sex, birth order (first, second, third or later), childcare environment (attending nursery school or not), bottle feeding (weaned or ongoing), daily snacking frequency (never, once or twice, three times or more), eating before bed (never, weekly, daily), wake-up time (before 7:00, 7:00–8:00, after 8:00, or unsettled), bedtime (before 21:00, 21:00–22:00, after 22:00, or unsettled), topical fluoride application (yes or no), and use of fluoride-containing toothpaste (yes or no). Among the 10 child-related items, sex, birth order, childcare environment, bottle feeding, snacking frequency, eating before bed, and bedtime were selected based on our previous study [17]. By contrast, wake-up time, topical fluoride application, and the use of fluoride-containing toothpaste were newly included to capture additional lifestyle and oral-health-related behaviors that may influence caries risk among long-term breastfed children. In this study, prolonged breastfeeding was defined as breastfeeding for 18 months or longer. This threshold was chosen because previous studies have used widely varying definitions of long-term breastfeeding, and evidence regarding children who continue breastfeeding beyond 18 months remains limited. By focusing on a more homogeneous group of long-term breastfed children, we aimed to identify factors associated with caries development within this specific population rather than compare different feeding modes or breastfeeding durations.

Information collected about the mothers included six items: feeling exhausted with parenting (yes or no), enjoying parenting (yes or no), anxiety (yes or no), physical condition (healthy or unhealthy), participation in prenatal classes (yes or no), and participation in postnatal oral health classes (yes or no). Among these six mother-related items, feeling exhausted with parenting, enjoying parenting, anxiety, and physical condition were selected based on our previous study [17]. Participation in prenatal classes and participation in postnatal oral health classes were newly added to capture maternal educational exposure and oral-health-related support that may influence children’s oral health behaviors. In Toyonaka City, prenatal classes are held for pregnant women to provide dietary and oral health guidance during pregnancy, while postnatal oral health classes are offered for mothers and their 8- to 10-month-old children to provide dietary and toothbrushing instruction, as well as dental consultations for the child. These classes are conducted only on weekdays.

### 2.5. Statistical Analyses

For descriptive analyses, relative and absolute frequencies, means, and standard deviations were calculated from univariate analyses. The number of erupted teeth, dmft, Cariostat scores, and body mass index (BMI) were compared between the caries-free group and the caries-experienced group using Student’s *t*-test. A logistic regression analysis was performed to evaluate caries risk factors, calculating odds ratios (ORs) and their 95% confidence intervals (CIs), with caries occurrence at 42 months as the dependent variable. All variables were entered simultaneously into the model using the forced-entry method in an exploratory manner. This approach was chosen because no established theoretical models or prior evidence specifically address risk factors among long-term breastfed children. To ensure the appropriateness of including all variables, multicollinearity was assessed using the variance inflation factor (VIF). Model fit was further evaluated using the Hosmer–Lemeshow goodness-of-fit test. All analyses were conducted using IBM SPSS Statistics version 28.0.1.0^®^ (IBM Japan, Tokyo, Japan). A *p*-value of < 0.05 was considered statistically significant.

## 3. Results

### 3.1. Characteristics of the Participants

The characteristics of the participants are shown in Table 1. Of the 1210 children, 294 (24.3%) had experienced dental caries at 42 months of age. In the caries-experienced group, the mean dmft at 18 months was 0.36 ± 1.3, and that at 42 months was 3.3 ± 2.6. No significant differences were observed in the number of erupted teeth or BMI at 18 months between the caries-free and caries-experienced groups. However, the caries-experienced group had a significantly higher Cariostat score at 18 months than the caries-free group (*p* < 0.001).

### 3.2. Univariate Analysis

Table 2 shows the results of the univariate analysis of dental caries distribution among factors in children. Several factors were significantly associated with dental caries experience at 42 months of age, including Cariostat score at 18 months (++: *p* < 0.001; +++: *p* < 0.001), birth order (second: *p* < 0.001; third or later: *p* < 0.001), snacking frequency at 18 months (once or twice: *p* < 0.001; ≥3 times: *p* = 0.011), wake-up time at 18 months (before 7:00: *p* < 0.001), bedtime at 18 months (before 21:00: *p* = 0.008), and use of fluoride-containing toothpaste at 18 months (*p* = 0.034). Among maternal factors, feeling exhausted with parenting when the child was 18 months old (*p* = 0.035), anxiety when the child was 18 months old (*p* = 0.049), and participation in postnatal oral health classes (*p* < 0.001) were also significantly associated with dental caries experience at 42 months of age (Table 3).

### 3.3. Logistic Regression Analyses

The results of the logistic regression analyses for dental caries experience at 42 months of age are presented in Table 4. After adjustment for potential confounders, several factors were significantly associated with dental caries experience at 42 months, including Cariostat score (++) (OR = 1.93; 95% CI: 1.32–2.82, *p* < 0.001), Cariostat score (+++) (OR = 5.09; 95% CI: 2.41–10.75, *p* < 0.001), being a second child (OR = 1.80; 95% CI: 1.29–2.53, *p* < 0.001), being a third or later child (OR = 2.29; 95% CI: 1.51–3.45, *p* < 0.001), snacking once or twice per day (OR = 1.52; 95% CI: 1.02–2.25, *p* = 0.038), wake-up time before 7:00 (OR = 0.63; 95% CI: 0.43–0.91, *p* = 0.015), feeling exhausted with parenting (OR = 1.51; 95% CI: 1.02–2.25, *p* = 0.041), and participation in postnatal oral health classes (OR = 0.56; 95% CI: 0.37–0.84, *p* = 0.005). The regression model demonstrated good overall fit according to the Hosmer–Lemeshow test (*p* = 0.733), and all VIF values were below the commonly accepted threshold (<4.0), indicating no problematic multicollinearity among the independent variables and supporting the stability of the estimates.

## 4. Discussion

Although the relationship between prolonged breastfeeding and ECC has not been consistently demonstrated and remains a subject of ongoing debate, dentists must respect mothers’ chosen feeding practices while managing children’s oral health and providing guidance on how to maintain oral health, allowing children to benefit from breast milk for as long as possible—particularly for mothers who wish to continue breastfeeding. However, to our knowledge, no studies have focused specifically on the characteristics of children who were breastfed long-term. The present longitudinal study highlights the importance of comprehensive social support for mothers in achieving both oral health and the continued benefits of breastfeeding for at least the first 18 months.

Of the children enrolled in this study, 24.3% had experienced dental caries at 42 months of age. According to recent data from Japan, the prevalence of dental caries at 3 years of age ranges from 14.6% to 20.0%, which is lower than in the present study [19,20,21]. Previous studies have suggested that children who are breastfed for prolonged periods are more likely to experience dental caries [8,9,10]. However, a recent systematic review concluded that breastfeeding for less than 24 months is not associated with an increased risk of ECC [10], and a longitudinal study reported that a higher intake of free sugars among breastfed children—rather than breastfeeding itself—was associated with an increased risk of ECC [22]. This finding is consistent with another cohort study showing that exclusive breastfeeding for 6–17 months exerts a protective effect against ECC [12]. Additionally, a systematic review identified nocturnal breastfeeding as one of the strongest risk factors for ECC [8]. In the present study, these two important risk factors—free sugar intake and nocturnal breastfeeding—could not be evaluated in breastfed children. Therefore, further research is warranted to examine the amount and frequency of free sugar consumption, as well as the duration and frequency of nocturnal breastfeeding, among breastfed children.

In long-term breastfed children, birth order was significantly associated with dental caries experience at 42 months of age in the present study. Later-born children may be influenced by their older siblings and exposed to free-sugar-containing foods earlier in childhood [23]. This suggests that later-born children may more readily develop snacking habits and higher plaque acidity. In other words, birth order, snacking habits, and plaque acidity—identified as risk factors for ECC in both the present and previous studies [17]—may be interconnected through dietary patterns. Future cluster analyses would be useful to clarify the relationships among these risk factors for ECC. By contrast, snacking three or more times per day at 18 months of age was not a significant risk factor for dental caries experience at 42 months. Considering this result, snacking habits that develop after 18 months of age, rather than those present at 18 months, may have a greater association with caries development at 42 months.

Waking up before 7:00 was negatively associated with ECC over time among long-term breastfed children in the present logistic regression analysis. Similarly, going to bed before 21:00 was also negatively associated in the univariate analysis. To our knowledge, no studies to date have examined wake-up time itself as a primary caries risk factor. Previous population studies and systematic reviews have reported that irregular or late bedtimes and shorter sleep duration may be risk factors for ECC [24,25]. The present findings may therefore suggest that establishing regular daily rhythms and healthy sleep habits by 18 months of age may be important for maintaining oral health in long-term breastfed children. However, research on the relationship between circadian rhythm development and nocturnal breastfeeding remains inconsistent [26,27,28], and nocturnal breastfeeding does not necessarily lead to an irregular daily rhythm or increased risk of ECC.

Maternal exhaustion with parenting when the child was 18 months of age was significantly associated with dental caries experience at 42 months among long-term breastfed children in the present study. Few studies have examined the association between chronic maternal stress and children’s dental caries experience [15]. Although the mechanisms remain unclear, several hypothetical pathways have been proposed. One possible explanation involves the quality of breast milk. It has been suggested that maternal stress may significantly reduce the amount of immunoglobulin A in breast milk [29]. In addition, psychosocial stress may affect mammary epithelial cells and secretory mechanisms, potentially altering the production and secretion of lactoferrin and lysozyme [30]. A decrease in these antibacterial components of breast milk could hypothetically contribute to the development of ECC in long-term breastfed children. Another plausible mechanism is behavioral: parental exhaustion may lead to less consistent toothbrushing and dietary management beyond breastfeeding, as parenting stress has been associated with reduced quality of childcare [31]. Therefore, these proposed pathways should be regarded as hypotheses to be tested, and further studies are needed to verify whether supporting maternal psychological well-being may help maintain oral health in long-term breastfed children [32].

A key strength of the present study is that it demonstrated, through a large-scale longitudinal design, that participation in postnatal oral health classes was negatively associated with ECC among long-term breastfed children. Previous studies have noted that prenatal and/or postnatal oral health support provided by dental professionals or trained non-professionals may benefit both maternal and infant oral health [13,14]. In the city where this study was conducted, postnatal oral health classes are offered for mothers and their children aged 8–10 months to provide dietary and toothbrushing instruction, as well as dental consultations for the child. These classes may help increase mothers’ interest, awareness, and motivation in caring for their children’s oral health. However, because the classes are only held on weekdays, participants are often mothers who are already highly motivated and have sufficient time to attend. Therefore, both the positive effects of the classes and the participants’ backgrounds may have influenced the present findings. Postnatal oral health instruction for mothers, as a public health measure, is important for maintaining children’s oral health while allowing them to benefit from breastfeeding as much as possible.

The present study has several limitations. First, our inclusion criteria limited the sample to children who continued breastfeeding for at least the first 18 months. This design inevitably excludes children with shorter breastfeeding duration, and may therefore introduce selection bias when considering the general population. Additionally, the definition of prolonged breastfeeding is not definitive and varies across studies [7,8,9,10,11,12]. Furthermore, no information was available on whether breastfeeding was exclusive or complementary, daytime or nighttime, or on its rate and frequency. These factors are strongly associated with ECC and may also correlate with some of the exposures included in our model. Therefore, the association between ECC and breastfeeding—particularly nighttime breastfeeding—could not be determined, and the adjusted ORs reported in this study should be interpreted with caution because the absence of these essential confounders may have led to residual confounding, potentially overstating or destabilizing some associations. Nevertheless, we believe that our findings provide a basis for further research to evaluate the actual conditions of long-term breastfed children. Second, the lack of quantitative information on free sugar intake and details about snack types made it difficult to fully separate breastfeeding from diet as a factor in caries development. The inclusion of dietary questionnaires or daily snacking records would likely have improved the explanatory power of the associations between breastfeeding and total sugar intake. Third, the reliability and validity of the questionnaire used have not been evaluated. In particular, the psychometric variables lacked established reliability, and responses may have been influenced by biases such as reporting or selection bias. Additionally, some continuous variables were categorized, potentially reducing the precision of the data. Other key confounding factors—such as socioeconomic status, parental education level, and oral hygiene habits—were also not included in the questionnaire, possibly affecting the results of this study. Fourth, although several variables reached statistical significance in the logistic regression analyses, the magnitude of their adjusted ORs (e.g., 1.52 for snacking, 1.51 for parental exhaustion, and 0.63 for early wake-up time) indicates only weak associations. Therefore, these findings should be interpreted cautiously, as their clinical relevance may be limited. These variables may serve as preliminary or hypothesis-generating factors rather than strong predictors of caries. Fifth, although the city’s socioeconomic indicators, such as income and education levels, are close to the national average, the presence of well-organized health promotion services may limit the generalizability of these findings to regions with different public health infrastructures. Future research that addresses these limitations—specifically through detailed breastfeeding assessments, quantitative evaluations of free sugar intake, and validation of the questionnaire’s reliability and accuracy—will help advance understanding of oral health in long-term breastfed children.

## 5. Conclusions

Waking habits in early childhood, maternal psychological condition, and participation in postnatal oral health instruction were significantly associated with the occurrence of dental caries over time among long-term breastfed children.

## Figures and Tables

**Table 1:** Descriptive statistics.

Variable	Caries-Free (*n* = 916)	Caries-Experienced (*n* = 294)	*p*
Number of erupted teeth (18 mo)	14.5 ± 2.3	14.4 ± 2.5	0.545
dmft (18 mo)	0.0	0.4 ± 1.3	<0.001
BMI (18 mo)	15.9 ± 1.8	16.0 ± 1.8	0.874
Cariostat score (18 mo)	1.1 ± 0.5	1.3 ± 0.6	<0.001
dmft (42 mo)	0.0	3.3 ± 2.6	<0.001

Student’s *t*-test was used to compare two groups. mo: months of age, dmft: decayed, missing, and filled primary teeth, BMI: body mass index. Data are presented as mean ± standard deviation. All variables were complete (*n* = 1210) because participants with any missing data were excluded from the analysis.

**Table 2:** Distribution of dental caries according to child-related factors.

Variables	Category	Caries Prevalence *n* (%)	*p*
Caries (42 mo)		294/1210 (24.3)	
Caries (18 mo)		31/1210 (2.6)	
Sex	Female	142/596 (23.8)	
	Male	152/614 (24.8)	0.706
Body mass index (18 mo)	Underweight	88/377 (23.3)	0.424
	Ideal	133/518 (25.7)	
	Overweight	73/315 (23.2)	0.417
Number of erupted teeth (18 mo)	≤12	64/262 (24.4)	0.875
	13–16	224/935 (24.0)	
	≥17	6/13 (46.2)	0.075
Cariostat score (18 mo)	−	7/45 (15.6)	0.322
	+	212/972 (21.8)	
	++	56/158 (35.4)	<0.001
	+++	19/35 (54.3)	<0.001
Birth order	First	84/405 (20.7)	
	Second	140/506 (27.7)	<0.001
	Third or later	70/199 (35.2)	<0.001
Nursery (18 mo)	No	209/817 (25.6)	
	Yes	85/393 (21.6)	0.134
Bottle-feeding (18 mo)	Weaned	275/1143 (24.1)	
	Ongoing	19/67 (28.4)	0.426
Snacking (18 mo)	No	193/899 (21.5)	
	Once or twice	57/170 (33.5)	<0.001
	≥3 times	44/141 (31.2)	0.011
Eating before bed (18 mo)	No	166/726 (22.9)	
	Weekly	45/162 (27.8)	0.185
	Daily	83/322 (25.8)	0.307
Wake-up time (18 mo)	Before 7:00	63/368 (17.1)	<0.001
	7:00–8:00	152/547 (27.8)	
	After 8:00	51/189 (27.0)	0.831
	Unsettled	28/106 (26.4)	0.772
Bedtime (18 mo)	Before 21:00	45/262 (17.2)	0.008
	21:00–22:00	173/683 (25.3)	
	After 22:00	53/186 (28.5)	0.383
	Unsettled	23/78 (29.5)	0.427
Topical Fluoride Application (18 mo)	No	268/1067 (25.1)	
	Yes	26/143 (18.2)	0.071
Fluoride-containing toothpaste (18 mo)	No	172/643 (26.7)	
	Yes	122/567 (21.5)	0.034

Univariate analysis was used to compare each item. mo: months of age. All variables were complete (*n* = 1210) because participants with any missing data were excluded from the analysis.

**Table 3:** Distribution of dental caries according to maternal factors.

Variables	Category	Caries Prevalence *n* (%)	*p*
Exhausting with parenting (18 mo)	No	231/1000 (23.1)	
	Yes	63/210 (30.0)	0.035
Enjoying parenting (18 mo)	No	2/12 (16.7)	
	Yes	292/1198 (24.4)	0.539
Anxiety (18 mo)	No	251/986 (25.5)	
	Yes	43/224 (19.2)	0.049
Physical condition (18 mo)	Healthy	252/1073 (23.5)	
	Unhealthy	42/137 (30.7)	0.066
Participation in prenatal class	No	276/1111 (24.8)	
	Yes	18/99 (18.2)	0.141
Participation in postnatal oral health class	No	254/938 (27.1)	
	Yes	40/272 (14.7)	<0.001

Univariate analysis was used to compare each item. mo: months of age. All variables were complete (*n* = 1210) because participants with any missing data were excluded from the analysis.

**Table 4:** Logistic regression analysis of factors associated with dental caries occurrence.

Variables	Category	Adjusted Odds Ratio (95% CI)	*p*	VIF
Cariostat score (18 mo)	+	Ref.		1.013
	−	0.68 (0.29–1.58)	0.373	
	++	1.93 (1.32–2.82)	<0.001	
	+++	5.09 (2.41–10.75)	<0.001	
Birth order	First	Ref.		1.115
	Second	1.80 (1.29–2.53)	<0.001	
	Third or later	2.29 (1.51–3.45)	<0.001	
Snacking (18 mo)	No	Ref.		1.089
	Once or twice	1.52 (1.02–2.25)	0.038	
	≥3 times	1.27 (0.83–1.96)	0.273	
Wake-up time (18 mo)	7:00–8:00	Ref.		1.052
	Before 7:00	0.63 (0.43–0.91)	0.015	
	After 8:00	0.98 (0.65–1.49)	0.930	
	Unsettled	0.95 (0.57–1.59)	0.850	
Exhausting with parenting (18 mo)	No	Ref.		1.238
	Yes	1.51 (1.02–2.25)	0.041	
Participation in postnatal oral health class	No	Ref.		1.135
	Yes	0.56 (0.37–0.84)	0.005	

All variables were entered simultaneously into the model using the forced-entry method. Only significant variables are shown. Adjusted for sex, birth order, eating before bed, snacking, bottle-feeding (18 mo), bedtime (18 mo), wake-up time (18 mo), exhausted with parenting (18 mo), enjoying parenting (18 mo), anxiety (18 mo), physical condition (18 mo), nursery attendance (18 mo), topical fluoride application (18 mo), use of fluoride-containing toothpaste (18 mo), participation in prenatal class, participation in postnatal oral health class, number of erupted teeth (18 mo), and Cariostat score (18 mo). mo, months of age; CI, confidence interval; VIF, variance inflation factor.

## Data Availability

The original contributions presented in this study are included in the article. Further inquiries can be directed to the corresponding author.

## Abbreviations

The following abbreviations are used in this manuscript:

BMI	body mass index
CI	confidence interval
ECC	early childhood caries
OR	odds ratio
VIF	variance inflation factor
WHO	World Health Organization
